# Targeting STEAP1 as an anticancer strategy

**DOI:** 10.3389/fonc.2023.1285661

**Published:** 2023-10-16

**Authors:** Hajime Nakamura, Yohei Arihara, Kohichi Takada

**Affiliations:** Department of Medical Oncology, Sapporo Medical University School of Medicine, Sapporo, Japan

**Keywords:** STEAP1, antibody therapy, CAR-T therapy, cancer vaccine, anticancer strategies

## Abstract

Although the six-transmembrane epithelial antigen of prostate 1 (STEAP1) was first identified in advanced prostate cancer, its overexpression is recognized in multiple types of cancer and associated with a poor prognosis. STEAP1 is now drawing attention as a promising therapeutic target because of its tumor specificity and membrane-bound localization. The clinical efficacy of an antibody-drug conjugate targeting STEAP1 in metastatic, castration-resistant, prostate cancer was demonstrated in a phase 1 trial. Furthermore, growing evidence suggests that STEAP1 is an attractive target for immunotherapies such as chimeric antigen receptor-T cell therapy. In this review, we summarize the oncogenic functions of STEAP1 by cancer type. This review also provides new insights into the development of new anticancer strategies targeting STEAP1.

## 1 Introduction

Cancer is still a major burden of disease worldwide despite the development of new therapeutic strategies. An estimated 19.3 million new cases and almost 10 million deaths have reportedly occurred due to cancer in 2020 (1). Further efforts are needed to prolong the survival time of patients with cancer, especially at the advanced stages.

The six-transmembrane epithelial antigen of prostate 1 (STEAP1), first identified in advanced prostate cancer, is a cell surface protein that functions as a transporter (2). STEAP1 is reportedly overexpressed in a subset of human cancers (3, 4). The Gene Expression Profiling Interactive Analysis (GEPIA) web server, which contains 9,736 tumors and 8,587 normal samples from the Cancer Genome Atlas (TCGA) and Genotype-Tissue Expression (GTEx) projects, also revealed high expression of STEAP1 in multiple cancer tissues compared to normal tissues (**Figure S1**) (5). Thus, considering its cell surface location and cancer specificity, STEAP1 is regarded as a promising therapeutic target in cancer. DSPT3068S, an antibody-drug conjugate (ADC) -targeting STEAP1, has been investigated in a phase I trial for patients with metastatic castration-resistant prostate cancer (6). Furthermore, a recent study demonstrated the development of a chimeric antigen receptor (CAR) against STEAP1, which showed remarkable efficacy against prostate cancer cells both *in vitro* and *in vivo* (7). Thus, STEAP1 is rapidly gaining attention for the development of novel treatment strategies of cancer.

In this review, we initially focus on the molecular mechanisms and functions of STEAP1 by cancer type and subsequently present several potential therapeutic strategies targeting STEAP1 based on previous pre-clinical and clinical reports.

## 2 Molecular mechanisms and functions of STEAP1

The STEAP family contains four members, named STEAP1–4, all of which have in common a six transmembrane domain with the COOH- and N-terminals located in the cytosol (8). STEAP1 was the first member of the STEAP family to be identified and has been widely studied as a gene related to cancer progression. STEAP1 was previously predicted not to promote iron and cooper reduction or uptake mainly due to the lack of the N-terminal NADPH-binding F420H2:NADP+ oxidoreductase domain unlike other STEAP members. However, a recent study revealed that STEAP1 exhibits cellular ferric reductase activity by fusing to the intracellular NADPH-binding domain of STEAP4. These findings can ultimately contribute to the development of STEAP1 targeted therapy (9). In contrast, the pathological functions of STEAP1 in cancer still need further investigation. In this section, we discuss the molecular mechanisms and functions of STEAP1 in a cancer-type-dependent manner.

### 2.1 Prostate cancer

STEAP1 was first identified as a prostate-specific-cell surface-antigen that is highly expressed in human prostate cancer (2). STEAP1 is reportedly expressed in more than 80% of the cases of metastatic castration-resistant prostate cancer with bone or lymph node involvement and its expression is elevated in all stages of the disease (10). A high level of STEAP1 expression was positively associated with Gleason scores, which is the most reliable histological grading for prostate cancer, and poor prognoses, suggesting that STEAP1 is involved in tumor initiation and progression (11, 12). Knockdown of *STEAP1* induces apoptosis and inhibits proliferation in prostate cancer cells (13). Further, monoclonal antibodies against STEAP1 were found to inhibit intercellular communication *in vitro* and suppress proliferation of tumor xenografts in a xenograft model of prostate cancer (14).

In terms of diagnosis, immunohistochemical analysis of prostate cancer specimens with a range of Gleason scores revealed that STEAP1 could be a suitable candidate to distinguish patients with cancer from patients without tumor (15). Furthermore, a recent study revealed that STEAP1-positive extracellular vesicle levels in plasma are significantly associated with prostate cancer diagnoses (16). The current standard diagnostic methods are prostate-specific antigen (PSA) screening and tissue biopsy, which have a high false-positive rate and are invasive, respectively (17, 18). Therefore, STEAP1 extracellular vesicles can be used for screening to improve the clinical management of prostate cancer.

Prostate cancer is thus the most investigated type of cancer with respect to STEAP1-targeted therapy. However, the underlying mechanisms of proliferation and invasiveness triggered by STEAP1 are still controversial, and little is known about the underlying pathways related to STEAP1 in prostate cancer compared to other types of cancer. Further efforts are warranted to clarify the STEAP1-related pathway to develop STEAP1-targeted treatment strategies in prostate cancer.

### 2.2 Colorectal cancer

**Figure 1:**
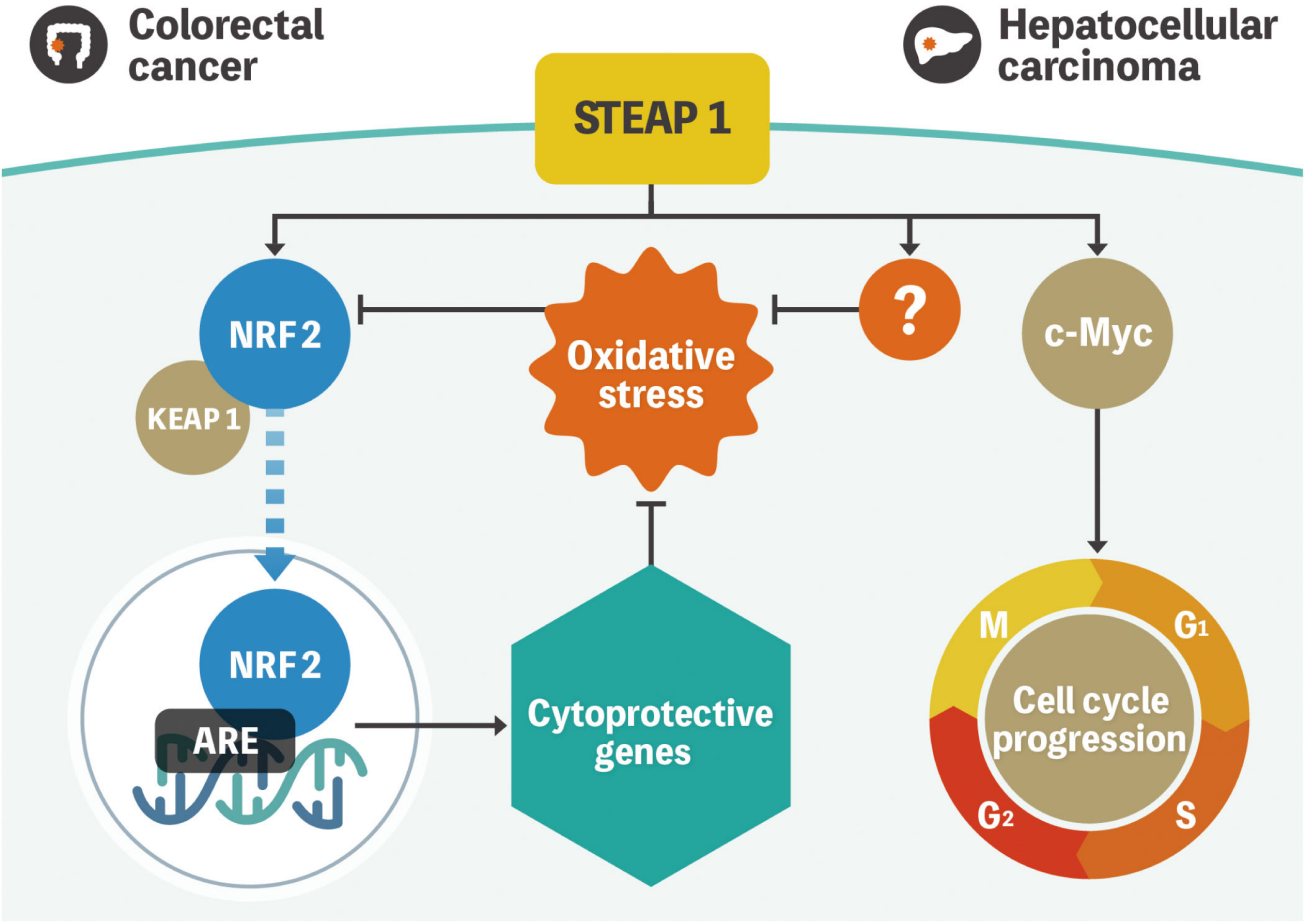
Model representation of STEAP1-mediated gene regulation in colorectal cancer and hepatocellular carcinoma. NRF2 is located in the cytoplasm and is regulated by KEAP1 under normal conditions. Oxidative stress triggers NRF2 dissociation from KEAP1 and subsequent translocation to the nucleus to activate multiple cytoprotective genes. STEAP1 seems to play a role in the activation of the pathway in colorectal cancer. Conversely, STEAP1 modulates ROS levels, although the role of the STEAP1-NRF2 axis is uncertain in hepatocellular carcinoma. In addition, STEAP1 promotes cell cycle progression by regulating the expression of c-Myc. STEAP1, the six-transmembrane epithelial antigen of prostate 1; NRF2, nuclear factor-erythroid 2-related factor 2; KEAP1, kelch-like ECH-associated protein 1; ROS, reactive oxygen species.

We have previously reported that STEAP1 is overexpressed in colorectal cancer (CRC) cells compared with the normal counterparts (19). Knockdown of *STEAP1* evoked intrinsic apoptosis in several CRC cell lines. We also measured intracellular ROS using flow cytometry to assess whether the knockdown of *STEAP1* stimulated ROS generation in CRC cell lines. Contrary to the results mentioned above, an increased generation of intracellular ROS was observed in CRC cell lines such as DLD-1 and SW480. *N*-Acetyl-cysteine, a ROS scavenger, suppressed the increase in ROS production and the intrinsic apoptosis evoked by *STEAP1* silencing. We also found that the expression of several nuclear factor-erythroid 2-related factor 2 (NRF2)-mediated antioxidant molecules, including heme oxygenase 1 (HMOX1), NAD(P)H quinone dehydrogenase 1 (NQO1), and thioredoxin reductase 1 (TXNRD1), was significantly down regulated by *STEAP1* silencing. Under normal circumstances, kelch-like ECH-associated protein 1 (KEAP1) binds to NRF2 and retains it in the cytoplasm. However, NRF2 dissociates from KEAP1 and translocates to the nucleus to activate downstream antioxidant enzymes (20, 21). Immunocytochemical analysis revealed that the nuclear translocation of NRF2 was inhibited by *STEAP1* silencing in DLD-1 cells. A significantly positive relationship between STEAP1 and NRF2 was also detected by performing Pearson’s correlation coefficient analysis on publicly accessible gene expression profiling data. These findings suggest that the STEAP1-NRF2 axis may be beneficial as a novel strategy to combat CRC (**Figure 1**).

### 2.3 Hepatocellular carcinoma

We had also previously investigated the biological mechanisms of STEAP1 in hepatocellular carcinoma (HCC). Similar to CRC, STEAP1 is overexpressed in HCC and associated with poor prognoses. The knockdown of *STEAP1* triggered G1 arrest, leading to the inhibition of cell proliferation in HCC. The results of bioinformatic analysis indicated that c-Myc, which is known to contribute to the pathogenesis of a broad range of human cancers, lies downstream of STEAP1, and the pathway promotes cell proliferation and cell-cycle progression in HCC. A recent study revealed that *STEAP1* silencing leads to increased phosphorylation of c-Myc in prostate cancer cells (22). Although there might be several types of STEAP1 pathways related to c-Myc, the underlying mechanisms still needs further investigation. In addition, intracellular ROS levels were also increased by STEAP1 inhibition, similar to CRC. However, we found no relationship between STEAP1 and NRF2 in HCC. Previous reports had demonstrated that c-Myc generates ROS in liver cancer cells, which is inconsistent with our findings (23, 24). These results suggest the existence of an NRF2- or c-Myc- independent ROS-related pathway in the regulation of STEAP1-mediated cell proliferation (**Figure 1**) (25).

### 2.4 Gastric cancer

Zhang et al. reported that STEAP1 was overexpressed and associated with poor prognoses in gastric cancer. Up-regulation of *STEAP1* increased cell proliferation, migration, and invasion via the AKT/FOXO1 pathway and caused epithelial–mesenchymal transition (EMT); these effects decreased after *STEAP1* silencing (26). Furthermore, Wu et al. revealed that STEAP1 is translationally induced during peritoneal metastasis and can drive both tumorigenesis and chemoresistance to docetaxel (27). These studies concluded that STEAP1 can be a potent candidate for targeted therapy.

### 2.5 Lung cancer

Several groups have reported that *STEAP1* is significantly upregulated in lung cancer compared to the normal cells and is associated with poor prognoses (28, 29). Gene Ontology (GO) and Kyoto Encyclopedia of Genes and Genomes (KEGG) analyses also revealed that *STEAP1* upregulation potentially regulates tumor progression via homologous recombination, p53 signaling, cell cycle, DNA replication, and apoptosis (24). Furthermore, knockdown of *STEAP1* using siRNA reduced endothelial cell migration and tube formation, which implicated STEAP1 as a novel vascular target in lung cancer (30). Importantly, STEAP1 regulates EMT via the Janus kinase 2 (JAK2)/signal transducer and activator of transcription 3 (STAT3) signaling pathway that is often detected in various tumors and involved in oncogenesis (31). Collectively, STEAP1 appears to be a promising biomarker and therapeutic target in lung cancer.

### 2.6 Ewing’s sarcoma

Ewing’s sarcoma (EWS) is the second most common bone cancer in children. Despite recent advances in the multimodal treatment for EWS, the 5-year survival rate is less than 30% in patients with metastases (32). STEAP1 has emerged as a target for EWS therapy. In EWS cells, overexpressed *STEAP1*, whose expression is induced by the EWS::FLI fusion gene binding to the *STEAP1* promoter lesion as a transcription factor, confers enhanced proliferative and invasive properties on the cells. STEAP1 induces the production of intracellular reactive oxygen species (ROS), followed by STAT1 activation (**Figure S2**) (33). In contrast, high membranous STEAP1 expression was correlated with improved overall survival (OS) in patients with EWS, which could be attributed to a correlation with sensitivity to chemotherapeutic agents such as doxorubicin and etoposide (34). Although these observations are valuable, they complicate decision-making while considering therapeutic interventions.

In addition to EWS::FLI1, NKX2.2 is a positive regulator of STEAP1 expression (35). Initially, the transcriptional repressive roles of NKX2.2 were recognized as being necessary and sufficient for the oncogenic properties of EWS (36). However, Markey et al. showed that NKX2.2 binds to two sites of the *STEAP1* promoter lesion, leading to increased STEAP1 expression. Moreover, by interacting with EWS::FLI1 at sites proximal to the EWS::FLI1 sites, NKX2.2 works as a co-regulator of STEAP1 expression (**Figure S2**). Thus, the elucidation of master transcriptional regulators of STEAP1 can help identify potential therapeutic targets of EWS.

### 2.7 Breast cancer

As mentioned earlier, STEAP1 confers oncogenic properties such as enhanced proliferation and metastatic potential and its high expression is a poor prognostic maker in a subset of cancers (37). In contrast, STEAP1 exerts tumor suppressive effects on breast cancer cells. *STEAP1* expression is downregulated in breast cancer tissues compared to normal cells, and low STEAP1 expression is associated with poor prognoses in patients with breast cancer (38). This can be attributed to EMT suppression by STEAP1 through *CDH1* upregulation and the downregulation of EMT-related genes in breast cancer cells. Researchers have also used bioinformatics analyses to demonstrate that low STEAP1 expression is related to poor prognoses (39). Therefore, interventions to inhibit STEAP1 can be useful as antimetastatic drugs for breast cancer.

## 3 Development of novel therapies by targeting STEAP1

STEAP1 is rapidly gaining attention as a novel therapeutic target because of its location on the cell surface and specificity to cancer versus normal cells. Of note, the functions of STEAP1 depend on the type of cancer. In this section, we discuss the development of STEAP1-targeting novel therapies.

### 3.1 Antibody therapy

DSTP3086S, a STEAP1-targeting ADC, has shown acceptable safety and potential benefit for patients with STEAP1-expressing, metastatic, castration-resistant, prostate cancer in a phase I trial (6). Eleven out of 77 patients treated with DSTP3086S once every 3 weeks met the response criteria of PSA reduction of ≥50%, while 26 out of 46 patients with evaluable disease at the baseline presented clinical response (two partial response; 24 stable disease). However, 69 out of 77 participants experienced an adverse event, with grade 3/4 noted in 24 participants, whereas no treatment-related deaths were observed in the study.

Bispecific T-cell engagers (BiTEs) are recombinant proteins made up of two single-chain variable fragments from two different antibodies, one targeting a tumor-specific antigen and the other targeting the effector T cell. Although these proteins have demonstrated dramatic therapeutic effects in patients with hematologic malignancies, they have not been investigated extensively for solid cancers (40, 41). Lin et al. demonstrated the efficacy of BC261, a rehumanized STEAP1-IgG, which is bispecific for STEAP1 and CD3 (42). BC261 reportedly showed significant elevation of T-cell infiltration and tumor ablation in EWS-family tumors and prostate cancer cell lines, confirmed by preclinical studies.

### 3.2 Chimeric antigen receptor -T cell therapy

CAR-T cell therapy is now one of the cutting-edge therapies for cancer. To date, six CAR-T therapies have been approved by the Food and Drug Administration (FDA) for the treatment of hematological malignancies such as lymphomas, some forms of leukemia, and multiple myeloma (43). In contrast to the results observed with hematologic malignancies, those from clinical trials of CAR-T cells targeting solid tumors were disappointing (44). However, Qi et al. recently reported the remarkable antitumor efficacy of anti-Claudin18.2 CAR-T for patients with gastric cancer, suggesting the potential benefit of CAR-T cell therapy for solid tumors (45).

**Figure 2:**
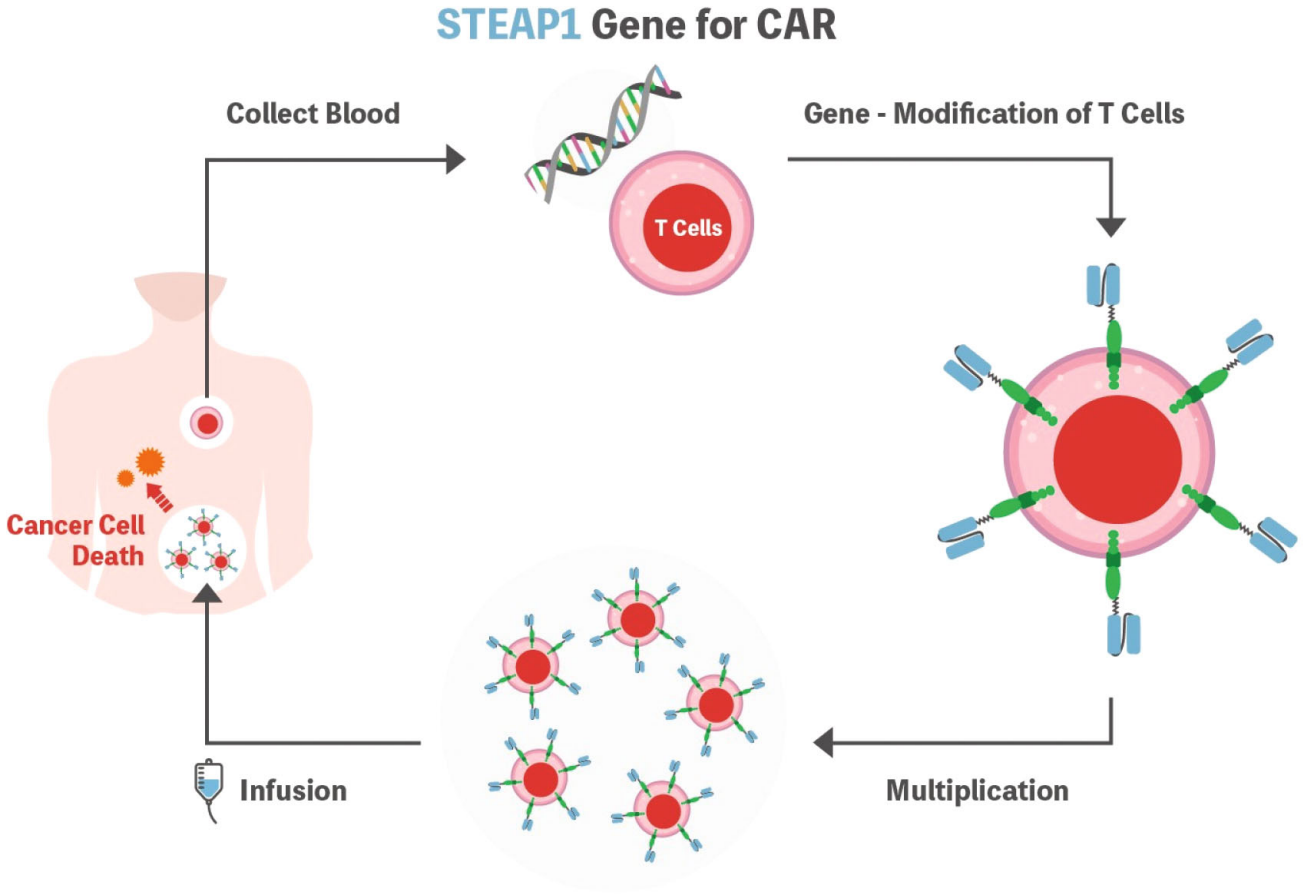
Schematic representation of the STEAP1 CAR-T cell. T cells are isolated from the patient’s blood and genetically modified to express CAR. Subsequently, cells are expanded ex vivo and then infused into the patient’s body to fight cancer cells. STEAP1 could be an attractive target as it is expressed in many cancer types. STEAP1, the six-transmembrane epithelial antigen of prostate 1; CAR, chimeric antigen receptor.

Considering the principles of CAR-T cell therapy, the most important aspect is identifying the tumor-specific antigen to be detected by the CAR, which should be highly expressed on the cell surface of the target but not on healthy human tissues. In this context, STEAP1 could be an attractive target as it is expressed in many cancer types, and high normal tissue expression of STEAP1 is only documented in the prostate, which is not a vital organ (**Figure 2**).

Although STEAP1 CAR-T has not yet been applied in the clinical trial setting, it showed promising anti-tumor activity in prostate cancer with CAR-T cell expansion and infiltration into the tumor microenvironment both *in vivo* and *in vitro* (7). In this preclinical study, the authors tested the second-generation anti-STEAP1 CAR with the 4-1BB co-stimulatory domain. The CAR-T product showed high transduction efficiency and polyfunctionality that were reported to be associated with clinical outcomes in anti-CD19 CARs (46). Furthermore, Bhatia et al. reported that STEAP1 CAR-T cells demonstrated reactivity in low antigen density, antitumor activity across metastatic prostate cancer models, and safety in a human STEAP1 knock-in mouse model (10). These preclinical results warrant further development of STEAP1 CAR-T for clinical trials.

### 3.3 Other immunotherapies

T cells expressing an engineered T cell receptor (TCR-T cells) have recently drawn attention as a novel immunotherapy for cancer, especially for solid tumors (47). Schirmer et al. successfully isolated a STEAP1^130/^HLA-A*02:01-peptide-restricted TCR. EWS cell growth was suppressed when treated with respective TCR tg CD8+ T cells compared to non-specific CD8+ T cells both *in vivo* and *in vitro* (48). A subsequent report from the group also revealed the local tumor control effect of TCR tg CD4+ T cells *in vivo* (49). Thus, STEAP1-specific TCRs could be useful for STEAP1-expressing tumors.

Furthermore, due to its specific overexpression in cancer tissues but not in normal tissues, STEAP1 is considered an attractive target for several immunotherapies, including cancer vaccines. Cancer vaccines for STEAP1 have been developed because several epitopes of STEAP1 are recognized by cytotoxic T lymphocytes (CTLs) and successfully evoke the activation of CTLs (50). Although some of these STEAP1-targeted vaccines were well tolerated and successfully evoked immunogenicity, these preclinical observations have yet to translate into clinical success (51, 52).

## 4 Discussion

STEAP1 has been recently investigated in a variety of tumor tissues in addition to prostate cancer. As mentioned in section 2, STEAP1 has an oncogenic role in multiple types of tumors such as prostate cancer, CRC, HCC, gastric cancer, and lung cancer. Conversely, a tumor-suppressive function has also been reported in some types of tumors such as EWS and breast cancer. These inconsistent results suggest multiple roles of STEAP1 in a cancer-dependent manner, which need to be investigated further. Based on our findings and reports from other groups, ROS could be one of the possible factors giving rise to this inconsistency. We previously reported that *STEAP1* silencing contributed to increased ROS levels, which resulted in cell apoptosis in CRC and HCC (19, 25). In contrast, *STEAP1* knockdown decreased ROS levels and *STEAP1* upregulation induced ROS production in EWS (33). In CRC, we hypothesized that the contribution of NRF2 to ROS manipulation was related to STEAP1. In EWS, STAT1 is reported to be a key regulator of ROS production induced by STEAP1. Collectively, it is assumed that multiple pathways exist between STEAP1 and ROS in a cancer-type specific manner. Furthermore, ROS has a dual role in cancer cell pathophysiology. At low to moderate levels, ROS act as signal transducers demonstrating the oncogenic properties in cancer cells. In contrast, excessive levels of ROS cause damage to cancer cells, leading to cell death. Therefore, evaluating the cell’s responsiveness to ROS depending on cancer types may also be crucial to assess the relationship between STEAP1 and ROS.

As discussed in section 3, STEAP1 is an ideal target of cancer treatment because it is located on the cell surface and is specifically expressed in various types of cancer. In this review, we mentioned different types of STEAP1 targeted therapy, including antibody therapy, CAR-T therapy, TCR-T therapy, and cancer vaccines. The results of these therapies are promising, and the diagnostic roles of STEAP1 such as in liquid biopsy in prostate cancer have also been reported recently (16). Although most of the research related to STEAP1 was conducted in prostate cancer, we believe that STEAP1 could also be an ideal target in other types of malignant tumors as growing evidence has revealed the overexpression of STEAP1 in cancerous cells compared to the normal counterpart. However, therapeutic or diagnostic strategies targeting STEAP1 have not been applied in clinical settings. Thus, large clinical trials are warranted in addition to further investigation of the role of STEAP1 at the molecular level.

## 5 Conclusion

In this review, we summarized the oncogenic functions of STEAP1 and several potential therapeutic strategies targeting STEAP1. Growing evidence has revealed that STEAP1 is an ideal target for cancer therapy. Further efforts are warranted to apply STEAP1 targeted therapy in a clinical setting.

## Author contributions

HN: Writing – original draft, Writing – review & editing. YA: Writing – original draft, Writing – review & editing. KT: Writing – original draft, Writing – review & editing.
