# Supplementary material for: Targeting STEAP1 as an anticancer strategy
Source: Front Oncol. 2023 Oct 16;13:1285661. doi: 10.3389/fonc.2023.1285661 (PMC10613890; doi:10.3389/fonc.2023.1285661)
Supplement: Supplementary file 1 [file DataSheet_1.docx]

Supplementary Material

## Supplementary Figures


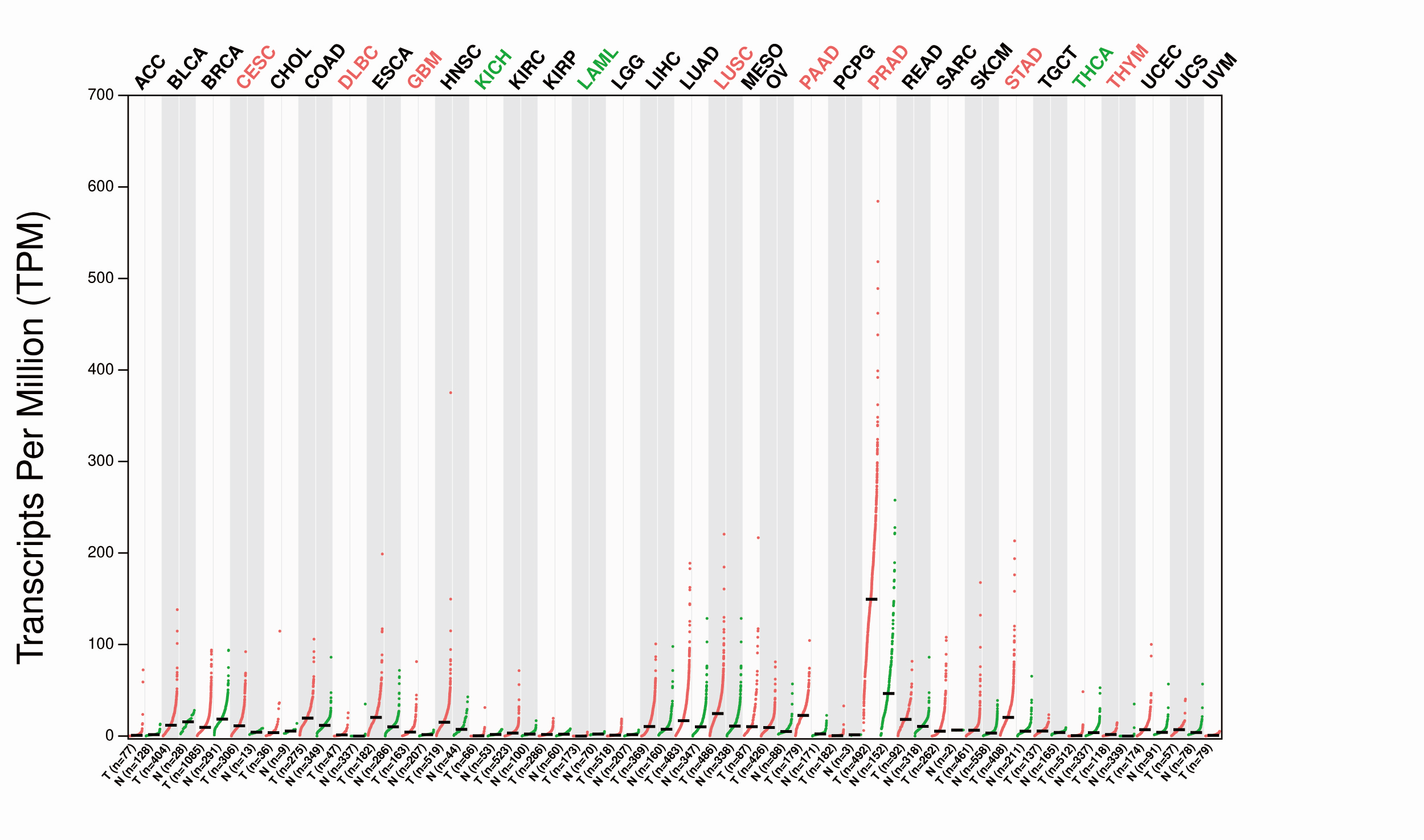


**Supplementary Figure 1.** The mRNA expression levels of STEAP1 in various types of tumor and normal tissues obtained from the GEPIA database. GEPIA, the Gene Expression Profiling Interactive Analysis; ACC, Adrenocortical carcinoma; BLCA, Bladder Urothelial Carcinoma; BRCA, Breast invasive carcinoma; CESC, Cervical squamous cell carcinoma and endocervical adenocarcinoma; CHOL, Cholangiocarcinoma; COAD, Colon adenocarcinoma; DLBC, Lymphoid neoplasm diffuse large B-cell Lymphoma; ESCA, Esophageal carcinoma; GBM, Glioblastoma multiforme; HNSC, Head and neck squamous cell carcinoma; KICH, Kidney chromophobe; KIRC, Kidney renal clear cell carcinoma; KIRP, Kidney renal papillary cell carcinoma; LAML, Acute myeloid leukemia; LGG, Brain lower grade glioma; LIHC, Liver hepatocellular carcinoma; LUAD, Lung adenocarcinoma; LUSC, Lung squamous cell carcinoma; MESO, Mesothelioma; OV, Ovarian serous cystadenocarcinoma; PAAD, Pancreatic adenocarcinoma; PCPG. Pheochromocytoma and Paraganglioma; PRAD, Prostate adenocarcinoma; READ, Rectum adenocarcinoma; SARC, Sarcoma; SKCM, Skin cutaneous melanoma; STAD, Stomach adenocarcinoma; TGCT, Testicular germ cell tumors; THCA, Thyroid carcinoma; THYM, Thymoma; UCEC, Uterine corpus endometrial carcinoma; UCS, Uterine Carcinosarcoma; UVM Uveal melanoma.


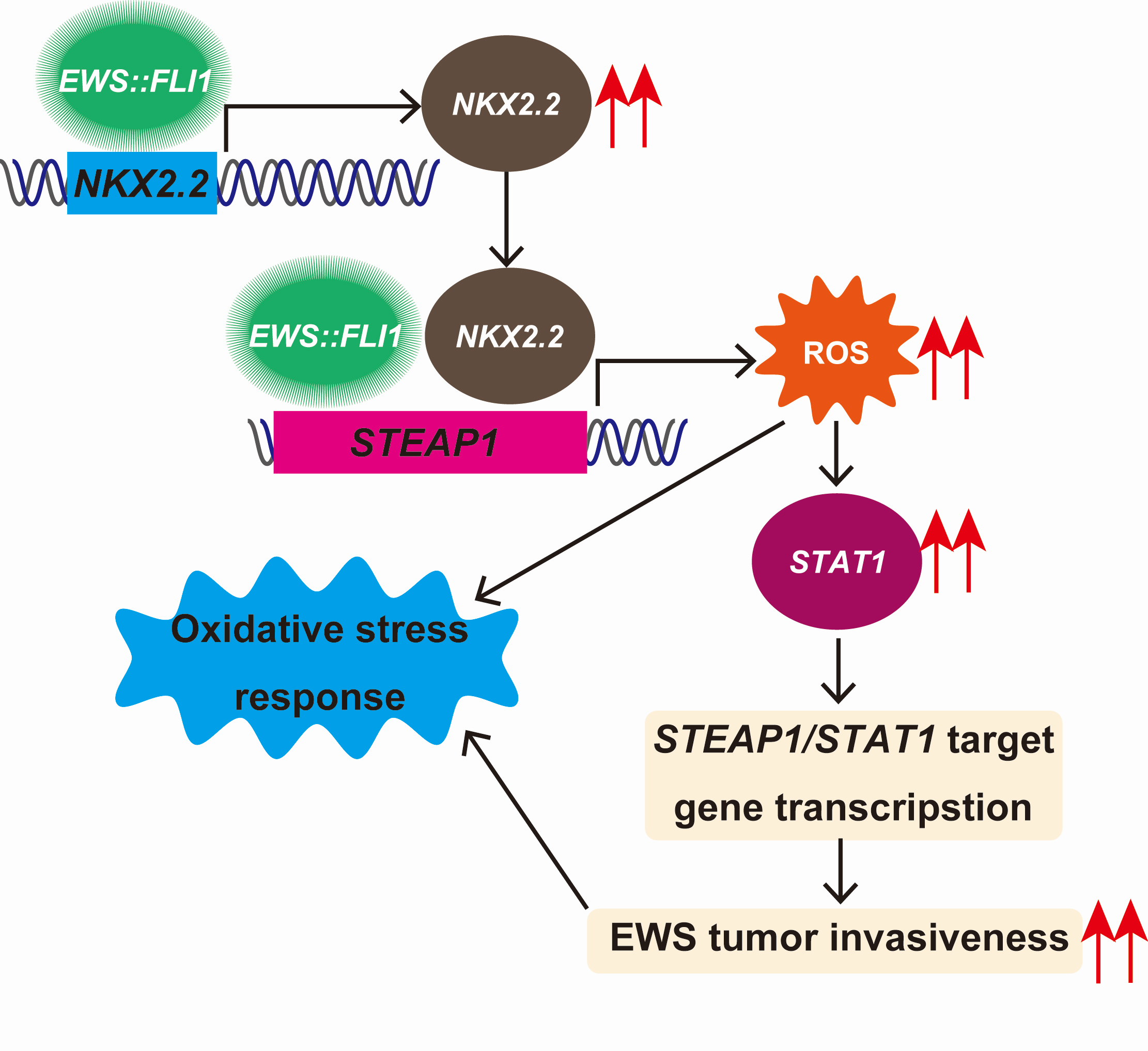


Supplementary Figure 2. Model representation of STEAP1-mediated gene regulation in EWS. EWS::FLI1 upregulates NKX2.2 by binding to the enhancer regions, leading to NKX2.2 binding to the STEAP1 promoter. EWS:FLI also binds to the STEAP1 promoter resulting in ROS production. STAT1 is upregulated by ROS production leading to increased EWS tumor invasiveness. EWS, Ewing cell sarcoma; STEAP1, the six-transmembrane epithelial antigen of prostate 1; NKX2,2, NK2 Homeobox 2; ROS, reactive oxygen species; STAT1, signal transducer and activator of transcription 1.

| **Table S1 Expression and survival impact of STEAP1 in human tumors** | | | |
| --- | --- | --- | --- |
| **Cancer type** | **Overexpression compared to normal counterpart** | **Association between high STEAP1 expression and prognosis** | **Citation** |
| Prostate cancer | Yes | poor | 2, 11, 12 |
| Colorectal cancer | Yes | poor | 19 |
| Hepatocellular carcinoma | Yes | poor | 25 |
| Gastric cancer | Yes | poor | 26 |
| Lung cancer | Yes | poor | 28, 29 |
| Ewing's sarcoma | Yes | good | 33 |
| Breast cancer | No | good | 38 |
